# Income-Based analysis of health security in Western Asia through an integrated GHSI, MCDM, and Clustering Model

**DOI:** 10.12688/f1000research.159002.1

**Published:** 2025-01-08

**Authors:** Adel A. Nasser, Abed Saif Ahmed Alghawli, Salem Saleh, Amani A. K. Elsayed

**Affiliations:** 1 Department of Information Systems and Computer Science, Sa’adah University, Sa’adah University, Sa'adah, Yemen; 2 Department of Artiﬁcial Intelligence,, Modern Specialized University, Sana'a, Yemen; 3 Department of Computer Science, College of Sciences and Humanities, Building No: 16 A 3,, Prince Sattam bin Abdulaziz University, Al Kharj, Riyadh Province, Saudi Arabia; 4 Department of Mathematics, Hodeidah University, Hodeidah, Yemen

**Keywords:** global health security, Western Asia, income, K-means clustering, CoCoSo, D-CRITIC weighting method, financial resource allocation

## Abstract

**Objectives:**

Infectious diseases present significant challenges to global health security in contemporary, interconnected global environments. This study aimed to evaluate and compare health security performance in Western Asia (WA), with a focus on income group-based disparities and region-specific insights.

**Methods:**

This study utilized the Global Health Security Index (GHSI) to assess health security across 17 WA countries categorized by income level. Health security indicators for 2019 and 2021 were analyzed using the D-CRITIC method to determine the relative importance of each indicator (Global Health Security Index, 2021): https://ghsindex.org/report-model/). A combined D-CRITIC-CoCoSo framework was employed to rank the countries, followed by K-means clustering for grading. The study also investigated correlations between financial allocation’s indicators and health security outcomes using Spearman’s rank correlation. A comparative analysis elucidated regional disparities across income categories.

**Results:**

This study highlights WA’s progress in health security by prioritizing foundational health systems, detection/reporting, rapid response, and risk management. From 2019 to 2021, priorities varied by income group, with high-income countries focusing on detection, upper-middle-income countries focusing on risk environments, and low-income countries focusing on prevention. While some nations demonstrated improvement, others, such as Armenia, experienced decline, revealing persistent vulnerabilities. This study revealed significant variability in health security capacity, with both progress and setbacks among countries in different clusters. High- and upper-middle-income countries, such as Qatar and Georgia, leverage investments and international partnerships to improve their rankings, while conflict-affected, low-resource countries, including Iraq, Yemen, and Syria, face stagnation or decline. Strong correlations were observed between financial resource allocation indicators and health performance. Higher investments in countries like Armenia and Georgia led to significantly improved health outcomes, while minimal spending in Syria and Yemen weakened their resilience to health threats.

**Conclusion:**

Disparities in health resilience persist, underscoring the need for equitable resource allocation and regional cooperation to enhance public health security.

## 1. Introduction

In today’s interconnected world, infectious diseases threaten global security, as seen with COVID-19, Ebola, and tuberculosis. These outbreaks disrupt economies, overwhelm healthcare, and affect vulnerable populations ( Berger et al., 2024; Chakaya et al., 2021). Surveillance data emphasize the rapid cross-border spread of disease ( Han et al., 2020), highlighting urgent gaps in preparedness and the need for unified international response strategies ( Khan et al., 2020). Enhancements in health security not only bolster a nation’s ability to respond to health crises but also promote global collaboration and trust among countries ( Bellm & Nuzzo, 2021). By improving surveillance systems, health infrastructure, and access to vaccines, nations can detect and manage outbreaks more effectively ( Nasser & Alghawli, 2024).

Western Asia emerges as a uniquely challenging region for health security, shaped by its intricate blend of geopolitical tensions, diverse populations, and varying health systems ( Roy & Quamar, 2022). With a history of political conflict and large-scale migration, the region requires robust health strategies to ensure accessible healthcare across borders ( Kalush & Lambert, 2023; World Health Organization, 2023). The effectiveness of health security systems here depends heavily on the region’s ability to monitor and respond swiftly to health crises, including infectious disease threats. Cross-border cooperation, resilient health infrastructure, equitable resource allocation, coordinated data-driven health security strategies, and policy interventions are essential pillars for addressing health risks ( Nasser & Alghawli, 2024). Therefore, evaluation and comparing Western Asia’s health security practices, identifying priorities, and setting improvement directions are critical for effectively managing public health across this complex region.

The Global Health Security Index (GHSI) serves as a crucial tool for assessing national health security, encompassing various aspects such as prevention, detection, and swift response capabilities ( Nasser & Alghawli, 2024; Bellm & Nuzzo, 2021).

In this paper, the first stage was analysing the current literature on the topic of study. Recent studies have made considerable progress in analysing and enhancing this index as a means to evaluate health security and public health resilience across countries, with numerous investigations highlighting its significance in shaping both national policies and global health security initiatives. For example, Boyd et al. conducted a study in 2020 examining the efficacy of the GHSI in measuring 195 nations’ readiness for biological threats, with a focus on how it might bolster future preparedness efforts. Assefa et al. (2020) investigated the connection between Global Health Security (GHS) and Universal Health Coverage (UHC) indices by utilizing Pearson’s correlation coefficient to measure the relationship between these two crucial global health metrics. That same year, Bezbaruah et al. (2021) explored the role of community health workers in reinforcing resilient health systems and health security. To demonstrate the need for additional funding, Yeh et al. (2021) delved into the risks and threats of infectious diseases in relation to the current political and socioeconomic contexts affecting GHS. Boyd et al. (2022) analysed the 2021 Global Health Security (GHS) Index Report, which indicates minimal improvement in global health security preparedness despite Worsley-Tonks et al. (2022) emphasizing the necessity to enhance disease surveillance worldwide, particularly in remote rural areas of low-income nations. A practical approach using Kenya as a case study was suggested. In 2020, Ravi et al. explored the practical applications of the GHS Index, potential uses to assist both professionals and policymakers in maximizing the tool’s utility, and the importance of ratings and rankings. Ledesma et al. (2023) employed indirect age normalization to assess the GHSI in order to examine the relationship between comparative mortality ratios of excess COVID-19 deaths and pandemic preparedness at the national level. Kumru et al. in 2022 studied the correlation between socioeconomic and demographic factors and COVID-19 mortality and morbidity rates in various countries. They also ranked the nations according to their COVID-19 rates. In 2024, Dobrovolska et al. utilized economic and mathematical modelling to examine the relationships between the GHSI and the Global Cyber security Index (GCSI) across 190 countries, with their findings underscoring the interdependencies between these indexes and illuminating potential synergies between health and cyber security. Concentrating on island nations, Boyd et al. (2024) investigated the connection between GHSI scores, excess mortality, and GDP per capita growth during the COVID-19 pandemic, underscoring the impact of health security on macroeconomic outcomes, especially in geographically isolated countries. Moreover, Ramírez-Soto and Arroyo-Hernández (2024) examined correlations between this index and MPOX case rates and discovered that high GHSI scores contribute to the early detection and response to outbreaks, reinforcing the index’s value in infectious disease preparedness.

However, while all those studies emphasized that the GHSI plays a significant role in assessing global preparedness and shaping both national policies and global health security initiatives, it exhibits several limitations, particularly when compared to more sophisticated tools such as multi-criteria decision-making (MCDM) models and machine learning clustering techniques.

One of its key shortcomings is the insufficient weighting of health security factors, which can lead to inaccurate rankings ( Nasser & Alghawli, 2024). Additionally, the absence of advanced ranking and clustering methods restricts the ability to differentiate between various subgroups, such as conflict versus non-conflict nations and countries with different income levels within the same region. These limitations, along with the lack of detailed comparative analysis or dynamic ranking clustering of nations, underscore the need for more nuanced methodologies that offer deeper insights into regional performance differences, hampering a comprehensive understanding of regional health security landscapes ( Nasser & Alghawli, 2024). To address these challenges, a more sophisticated framework that applies advanced methodologies and provides deeper insights into regional variation is essential. Several studies have made significant contributions to this regard. For instance, Sun et al. (2024) applied the fuzzy analytic hierarchy process algorithm to analyse the Global One Health Index in 160 countries and territories worldwide. Five additional studies leveraged multi-criteria decision-making (MCDM) methods to assess the 2019 and 2019 GHSI. Huang et al. 2021, employing modified VIKOR and CRITIC weighting, found its rankings diverged from other MCDM methods, suggesting VIKOR captures unique aspects of health system performance. Altıntaş 2022, using MAIRCA on EU member states, revealed strong correlations between GHSI scores and most other MCDM methods, except MAUT, indicating a broad agreement on relative health security performance. Pereira et al. 2022, utilizing PROMETHEE II and SMAA, coupled with a clustering approach, revealed inconsistencies between GHSI classifications and observed COVID-19 performance, highlighting potential flaws in the GHSI’s predictive capabilities and advocating for its revision. Nasser and Alghawli, 2024, proposed an entropy TOPSIS k-means clustering approach for ranking and clustering African countries’ health security practices. Nasser et al. 2024a applied entropy-VIKOR-K means clustering methods to determine and compare the relative importance of health security indicators in the EMR region in both 2021 and 2019 and to rank cluster countries based on their overall performance. This study also utilized Pearson’s and Spearman’s rank correlation coefficients to assess the relationships between the indicators and overall performance. However, in addition to the previously defined limitations of the traditional statistical methods used by the GHSI, these studies found limited applications of the GHSI in global health security assessment and emphasized the need for new evaluation methodologies ( Bulut et al., 2024; Nasser et al., 2024a; Nasser and Alghawli, 2024). Bulut et al. (2024) confirmed that methodologies that integrate MCDM with GHSI are very limited in the literature and have been used to a limited extent for the purpose of proposing new methodologies and ways to evaluate and rank countries’ performance, which is also in line with the findings of limited sophisticated ranking and clustering analyses that restrict the ability to compare health security across regions ( Nasser et al. 2024a).

In summary, although numerous studies have analyzed health security performance through diverse methodologies, none have integrated income classification, the Global Health Security Index (GHSI), Distance Correlation-based Criteria Importance through Inter-criteria Correlation (D-CRITIC) weighting, the Combined Compromise Solution (CoCoSo), and K-means clustering techniques specifically to examine health security in the Western Asian region. This study presents a novel approach as it introduces an innovative framework for analysing health security in Western Asia, integrating the Global Health Security Index (GHSI), income-based country classification, advanced multi-criteria decision-making (MCDM) methods, specifically D-CRITIC weighting, CoCoSo ranking, and K-means clustering. While prior research has explored health security performance using various approaches, no study has applied this specific combination, which enhances the accuracy through the collaborative strength of MCDM methods. Focusing on 2019–2021 data, this framework evaluates health security performance across Western Asia, emphasizing the differences between income groups. By utilizing D-CRITIC, this study determines the relative importance of health security indicators, while CoCoSo ranks countries based on overall performance, allowing for comparative insights across income classifications. K-means clustering further identifies distinct health security profiles, offering a more comprehensive understanding of the variance between countries, and finally, the Spearman rank correlation method further investigates the relationship between the average performances of financial resource allocation indicators and the COCOSO health security performance scores throughout the study period.

## 2. Methods

The methodology of this study followed a six-stage process. This section details each stage along with the integrated model used.

### 2.1. Data Acquisition: The Global Health Security Index (GHSI) as a Tool for Health Security Preparedness Assessment

The Global Health Security Index (GHSI) is a comprehensive instrument for assessing countries’ preparedness and capabilities in addressing health security threats. However, its suitability as a research tool warrants careful consideration given the discrepancies observed between predicted and actual performance during the COVID-19 pandemic ( Kaiser et al., 2021). The GHSI provides a multidimensional approach to evaluating health security, encompassing various aspects such as prevention (PR), detection, reporting (DR), rapid response (RR), health system capacity (HS), commitment and adherence (CA), and risk environment (RE) ( Wang and Lyu, 2023; Nasser et al., 2024a; Nasser and Alghawli, 2024). The GHSI’s publicly accessible data support extensive research opportunities and offer a holistic perspective on a country’s health security preparedness, and its structured approach facilitates robust comparisons among nations, rendering it a potentially valuable tool for our research ( Global Health Security Index, 2021). The World Bank classification, which categorizes countries into four income groups based on gross national income (GNI) per capita, delineates the 17 Western Asia (WA) countries as follows: Low-income countries (LIC) comprise Syria and Yemen; lower-middle-income countries (LMC) include Jordan and Lebanon; upper-middle-income countries (UMC) encompass Armenia, Azerbaijan, Georgia, Iraq, and Turkey; and high-income countries (HIC) consist of Bahrain, Cyprus, Israel, Kuwait, Oman, Qatar, Saudi Arabia, and the United Arab Emirates. For our comparative analysis, we extracted data for 17 countries in the WA region, including 2 LIC, 2 LMI, 5 UMC, and 8 HIC. To assess health security outcomes across these groups for each indicator, we examined performance metrics from 2019 and 2021 ( Global Health Security Index, Global Health Security Index Data Model and Report. (2021). Available at https://ghsindex.org/report-model/). Comprehensive data can be found in Tables 1-3 of the supplementary file ( Nasser, et al. 2024b) (available at: data repository https://doi.org/10.6084/m9.figshare.27992735.v3).

Furthermore, to assess the relationship between the financial resources allocated to public health preparedness and the effectiveness of resource utilization from one perspective and the health security outcomes in the region from another, this study utilizes GHSI data on two indicators: F1, a financing indicator, and F2, public healthcare spending levels per capita. A financing indicator, in the context of health security, is a metric that quantifies the financial resources dedicated to enhancing a country’s capacity to prevent, detect, and respond to disease outbreaks. This indicator provides a comprehensive representation of the financial investments made in various domains related to health security, such as preparedness funding, commitments based on Joint External Evaluation (JEE) and Performance Verification System (PVS) assessments, emergency response financing, and accountability for international commitments. The second indicator refers to the domestic general government health expenditure per capita. Comprehensive data can be found in Table 4 of the supplementary file ( Nasser, et al. 2024b) (available at: data repository https://doi.org/10.6084/m9.figshare.27992735.v3).

### 2.2. Applying the D-CRITIC Method to Determine and Compare the Relative Importance of Health Security Indicators across Western Asia and Its Income Groups

This study employed the modified version of the CRiteria Importance Through Inter-criteria Correlation (CRITIC) method, namely the Distance Correlation-based CRITIC (D-CRITIC) method to evaluate and compare the relative importance of health security indicators across Western Asia. D-CRITIC improves upon the original CRITIC method by incorporating distance correlation, enabling objective criteria weighting based on both variance and interdependency.

This approach was introduced by Krishnan et al. in 2021; it yields more valid and stable weighting and ranking results than traditional CRITIC, thus enhancing decision-making reliability ( Krishnan et al., 2021). D-CRITIC offers significant advantages for multi-criteria decision-making (MCDM). By considering both linear and nonlinear relationships between indicators through distance correlation, it enhances the accuracy of objective criteria weighting ( Duo et al., 2024). Its data-driven approach minimizes subjectivity and potential bias, leading to more equitable, stable, and valid outcomes compared to the CRITIC method ( Duo et al., 2024).

The procedure typically encompasses six distinct steps ( Krishnan et al., 2021):

Step 1. Creating a decision matrix (DM), denoted by $U_{\mathrm{ij}}$ as follows:

$$U_{\mathrm{ij}}=\begin{array}{ccc} u_{11} & u_{12} & \ldots .u_{1n}\\ \ldots & .. & \ldots .\ldots ..\\ u_{m1} & u_{m2} & \ldots .u_{\mathrm{mn}} \end{array}\;$$ (1)

The score of country i on indicator j is represented by $\;u_{\mathrm{ij}}$ . Additionally, the variables m and n indicate the total number of alternatives (countries) and criteria (health security indicators), respectively.

Step 2: Computing the normalized DM using (2):

$$z_{\mathrm{ij}}=\left\{ \begin{array}{c} \;\frac{u_{\mathrm{ij}}}{u_{j}^{+}},\;\text{for}\;\text{benefit criteria}\\ \frac{u_{j}^{-}}{u_{\mathrm{ij}}},\;\text{for}\;\text{cost criteria} \end{array}\right.$$ (2)

In this scenario, $u_{\mathrm{ij}}$ denotes the score assigned to alternative *i* for criterion *j*, $u_{j}^{+}$ represents the highest score attained for criterion *j*, and $u_{j}^{-}$ represents the lowest score recorded for criterion *j*.

Step 3: Calculating Standard Deviation ( *SD*_*j*_) for each criterion:

$${\mathrm{SD}}_{j}=\sqrt{\frac{\sum_{j=1}^{n}{\left(z_{\mathrm{ij}}-\bar{z_{j}}\right)}^{2}}{m-1}}\;$$ (3)

The symbol ( $\bar{z_{j}}$ ) represents the arithmetic mean (average) of the normalized values for criterion ‘ *j*’ across all ‘ *m*’ alternatives.

Step 4: Exploring Inter-Criterion Relationships with Distance Correlation

Equation (3) defines this calculation for any two criteria, represented as $c_{j}$ , and $c_{j^{\prime }}$ .

$$\text{dCor}\left(c_{j},c_{j^{\prime }}\right)=\frac{\text{dCov}\;\left(c_{j},c_{j^{\prime }}\right)}{\text{sqrt}\left(\text{dVar}\left(c_{j}\right)\;\text{dVar}\left(c_{j^{\prime }}\right)\right)}\;$$ (4)

Here, $\text{dCov}\;\left(c_{j},c_{j^{\prime }}\right)$ represents the distance covariance between $c_{j}$ and $c_{j^{\prime }}$ , while $\text{dVar}\left(c_{j}\right)=\text{dCov}\;\left(c_{j},c_{j}\right),$ represents the distance variance of $c_{j}$ , and $\text{dVar}\left(c_{j^{\prime }}\right)=\text{dCov}\;\left(c_{j^{\prime }},c_{j^{\prime }}\right)$ represents the distance variance of $c_{j^{\prime }}$ .

Step 5: Computing Criterion Information Content:

$$I_{j}={\mathrm{SD}}_{j}\sum_{j^{\prime }=1}^{n}\left(1-\text{dCor}\left(c_{j},c_{j^{\prime }}\right)\right)\;$$ (5)

The symbol $I_{j}$ represents a metric that quantifies the amount of information contained within the *j*th criterion.

Step 6: Compute the objective weights.

In this step, Equation (5) is used to calculate the objective weight of each jth criterion, reflecting its relative importance in decision-making.

$$w_{j}=\frac{I_{j}}{\sum_{j=1}^{n}I_{j}}$$ (5)

In our study, this process was repeated eight times to assess the relative importance of health security indicators in the WA, LIC-LMC, UMC, and HIC groups of countries in the region for both 2019 and 2021. Detailed calculations for this step are readily available in supplementary file (Sec. 2) ( Nasser, et al. 2024b) (Available at https://github.com/ProfAdelAbdulsalam/supplementary-material-and-softwares/tree/1.0.0) ( https://doi.org/10.5281/zenodo.14541236).

### 2.3 Assessing and ranking WA countries using a combined D-CRITIC-COCOSO framework

The Combined Compromise Solution (CoCoSo) method, introduced by Yazdani et al. (2019), is a robust tool in Multi-Criteria Decision-Making (MCDM). This method effectively integrates the strengths of simple additive weighting (SAW) and the exponentially weighted product model, making it particularly adept at generating balanced and reliable rankings. Its intuitive design, adaptability, and capacity to deliver actionable insights have made it widely applicable across various decision-making contexts, enhancing its relevance to our study ( Banihashemi et al., 2021; Naeem et al., 2022; Wang et al., 2023). It comprises the following implementation steps ( Yazdani et al. (2019):

• Step 1: Constructing Initial and Normalized Decision Matrices

In this step, the initial DM ( $U_{\mathrm{ij}}$ ) is constructed as described in (1) and then normalized as outlined in (6):

$$y_{\mathrm{ij}}=\left\{ \begin{array}{c} \;\frac{u_{\mathrm{ij}}-u_{j}^{-}}{u_{j}^{+}-u_{j}^{-}},C\;\text{is}\;a\;\text{benefit criteria}\\ \frac{u_{\mathrm{ij}}-u_{j}^{+}}{u_{j}^{-}-u_{j}^{+}},C\;\text{is}\;a\;\text{cost criteria} \end{array}\right.,$$ (6)

• Step 2. Computing the weighted comparability sequences, which are derived by (7) and (8):
$$S_{i}=\sum_{j=1}^{n}w_{j}y_{\mathrm{ij}},$$ (7)
$$P_{i}=\sum_{j=1}^{n}{\left(y_{\mathrm{ij}}\right)}^{w_{j}},$$ (8)

In this context, the weighting vector ( $w_{j}$ ) indicates the relative significance assigned to the *j*-th indicator, while ( $y_{\mathrm{ij}})$ represents the normalized performance score of the i-th alternative concerning the *j*-th indicator.

• Step 3: Calculating the evaluation scores of the alternatives

At this stage, three distinct evaluation scores are calculated as follows:

$$k_{\mathrm{ia}}=\left(P_{i}+S_{i}\right)/\sum_{i=1}^{m}\left(P_{i}+S_{i}\right),$$ (9)

$$k_{\mathrm{ib}}=\left(S_{i}/\min\;S_{i}\right)+\left(P_{i}/\min\;P_{i}\right),$$ (10)

$$k_{\mathrm{ic}}=\frac{\left(\lambda \left(S_{i}\right)+\left(1-\lambda \right)P_{i}\right)}{\left(\lambda \;\left(\max\;S_{i}\right)+\left(1-\lambda \right)\max\;P_{i}\right)},$$ (11)

Here, the parameter *λ*, ranging from 0 to 1, allows the decision-maker to fine-tune the relative importance of two aggregation techniques in determining the final compromise solution: the additive weighted sum ( *S*_*i*_) and the exponentially weighted product ( *P*_*i*_). This parameter serves as a control mechanism for balancing the influence of these two methods. A common practice is to use a value of 0.5, which gives equal weight to both *S*_*i*_ and *P*_*i*_, ensuring a balanced consideration of the two approaches in the final outcome. This equal weighting is often employed to achieve a balanced integration of both techniques in the ultimate result.

Step 4: Calculating the composite score index and rank alternatives

Finally, Equation (12) is used to calculate the composite score.

$$C_{i}=\left({\left(k_{\mathrm{ia}}+k_{\mathrm{ib}}+k_{\mathrm{ic}}\right)}^{\frac{1}{3}}\right)+\left(\frac{\left(k_{\mathrm{ia}}+k_{\mathrm{ib}}+k_{\mathrm{ic}}\right)}{3}\right),$$ (12)

In this investigation, the aforementioned process was iterated twice to rank Western Asian countries based on their GHSI scores for both 2019 and 2021. The alternatives are arranged in descending order according to their calculated scores. For a comparative analysis, Western Asian-ranked countries were further categorized by income group. Detailed calculations for this procedure are available in the supplementary file (Sec. 3) ( Nasser, et al. 2024b) (Available at https://github.com/ProfAdelAbdulsalam/supplementary-material-and-softwares/tree/1.0.0) ( https://doi.org/10.5281/zenodo.14541236)

### 2.4 Clustering Western Asian Countries Based on Their Composite Score Index

K-means clustering is a widely used learning algorithm for data mining and pattern recognition ( Chong 2021). It partitions n observations into k clusters, where each observation belongs to the cluster with the nearest mean. The algorithm operates by iteratively assigning data points to the closest cluster center and subsequently recalculating the cluster centers based on new assignments ( Cui et al., 2013).

This method is well known for its efficiency, simplicity, and adaptability across various fields. The advantages of K-means include computational efficiency and ease of implementation, rendering it suitable for large datasets ( Chong, 2021). The standard K-means algorithm follows these steps ( Cui et al., 2013): 1) initialize k cluster centers, often randomly; 2) assign each data point to the nearest cluster center; 3) recalculate the cluster centers based on the new assignments; 4) repeat steps 2 and 3 until convergence or a maximum number of iterations is reached.

In this study, the aforementioned process was also repeated twice to cluster Western Asian countries based on their D-CRITIC-COCOSO scores for both 2019 and 2021. The countries were clustered using a five-tiered grading system, ranging from “excellent” (Grade 1) to “poor” (Grade 5). The Western Asian-clustered countries were further divided into income tiers for comparative research. The supplementary file contains detailed calculations for this process (Sec.4) (Available at https://doi.org/10.6084/m9.figshare.27992735.v3) ( Nasser, et al. 2024b).

### 2.5 Examining the correlation between independent financial resource allocation indicators and D-CRITIC-COCOSO Health Security Outcomes

We utilized the Spearman rank correlation method to investigate the relationship between the average performances of financial resource allocation indicators (financing (F1), and public healthcare spending levels per capita (F2)) and the mean COCOSO health security performance scores (Ci) throughout the study period. This non-parametric technique is particularly appropriate for evaluating the association between two variables without assuming specific underlying distributions ( Liu, 2017). The Spearman correlation coefficient (ρ) was determined by independently ranking the sample values of both variables and then inserting the squared differences between these ranks into a formula derived from the Pearson correlation ( Zimmerman, 1994). This approach is beneficial as it emphasizes the relative positions of the values rather than their absolute disparities, making it resilient to outliers and nonlinear relationships ( Liu, 2017). An alternative method involves converting the values of both variables into standard scores, ranking the combined standard scores in a single sequence, and then calculating the Pearson correlation between the ranks corresponding to the original scores. This modified approach has been shown to be slightly more powerful than the conventional Spearman method for various distributions and sample sizes, ranging from 8 to 30 ( Zimmerman, 1994). In summary, the Spearman rank correlation method was chosen because of its ability to assess the strength and direction of the monotonic relationship between resource allocation indicators and COCOSO scores. This technique is particularly effective in situations where the relative rankings are more important than the exact values, making it well suited for comparing performance indicators across different domains ( Liu et al., 2010; Liu, 2017).

### 2.5 Comparative analysis

This phase consolidates and evaluates the outcomes from previous stages, examining the comparative importance of indicators, national rankings, and grouping trends across Western Asia and its economic subdivisions. The evaluation centered on uncovering regional inequalities and crucial patterns in health security performance for both 2019 and 2021. The outcomes will guide the creation of specific suggestions to improve health security readiness in each income category and the region overall. The key findings are outlined in the following sections.

### 2.6 Software

To determine the relative significance of health security indicators across Western Asian nations, we developed two advanced Excel-based software tools. Supplementary Software 1 - Distance Correlation-Based CRITIC Software. Source software available from (Available at https://github.com/ProfAdelAbdulsalam/supplementary-material-and-softwares/tree/1.0.0). Archived software available from ( https://doi.org/10.5281/zenodo.14541236) ( Nasser, et al., 2024b), License: OSI approved open license software is under GNU General Public License v3.0). This tool enables a detailed analysis by categorizing countries into income groups—Low-Income Countries (LIC), Lower-Middle-Income Countries (LMC), Upper-Middle-Income Countries (UMC), and High-Income Countries (HIC)—and provides valuable insights for the years 2019 and 2021. This tool specifically focuses on analyzing health security in low-income and lower-middle-income countries.

For the ranking analysis, we created Supplementary Software 2—An Integrated GHSI, MCDM, and Clustering Model for Health Security Analysis in Western Asia.

Source software available from: ( https://github.com/ProfAdelAbdulsalam/supplementary-material-and-softwares/tree/1.0.0). Archived software available from ( https://doi.org/10.5281/zenodo.14541236) ( Nasser, et al., 2024b), License: OSI approved open license software is under GNU General Public License v3.0). Supplementary Software 2 evaluates the health security performance of these nations for both 2019 and 2021, integrating weighting, ranking, clustering, and Spearman rank correlation analysis into a unified Excel-based tool, offering a robust framework for health security performance assessment.

For clustering analysis, the software Cluster Analysis for Marketing (2024) was utilized, which is available for free download at Cluster Analysis for Marketing - Free Download ( https://www.clusteranalysis4marketing.com/a-marketers-guide-to-cluster-analysis/free-download/).

## 4. Results and Discussion

### 4.1 Relative Importance of Health Security Indicators in WA region (2019 vs 2021)

**Figure 1.**
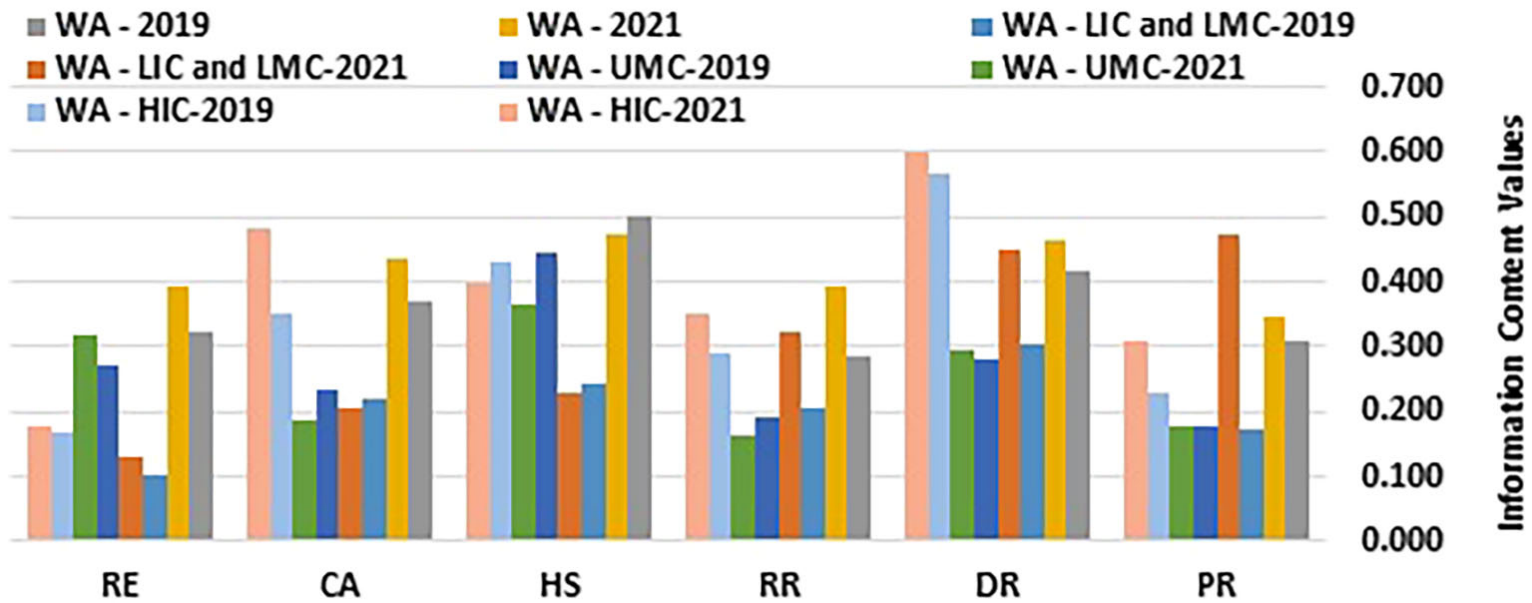
Information Content Values. (Source: Authors). This Figure chart illustrates the information content values for various health security-related dimensions, comparing them by income group and region over the years 2019 and 2021. The dimensions analyzed include Prevention (PR), Detection and Reporting (DR), Rapid Response (RR), Health Systems (HS), Commitments and Adherence (CA), and Risk Environment (RE). Data is presented for Western Asia (WA), stratified into Low-Income Countries (LIC), Lower-Middle-Income Countries (LMC), Upper-Middle-Income Countries (UMC), and High-Income Countries (HIC).

**Figure 2.**
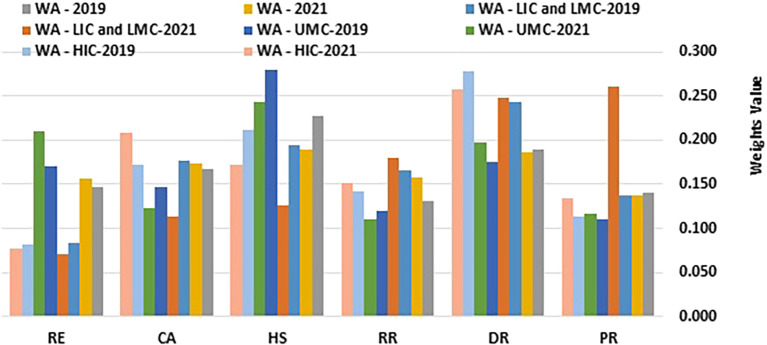
Weights values. (Source: Authors). This bar chart illustrates the weight values assigned to various health security-related dimensions, comparing them by income group and region over the years 2019 and 2021. The dimensions analysed include Prevention (PR), Detection and Reporting (DR), Rapid Response (RR), Health Systems (HS), Commitments and Adherence (CA), and Risk Environment (RE). Data is presented for Western Asia (WA), stratified into Low-Income Countries (LIC), Lower-Middle-Income Countries (LMC), Upper-Middle-Income Countries (UMC), and High-Income Countries (HIC).

Generally, in the D-CRITIC method, an indicator’s weight is directly linked to its information content (IC), with higher weights assigned to indicators that offer greater unique information, as measured by distance correlation. This adjustment ensures that indicators contributing more valuable information are weighted more heavily than those with less distinct content ( Krishnan et al., 2021). Table 1 and Figures 1 and 2 present the computed information content values and corresponding weights.

**Table 1.** Calculated information content values and weights of health security indicators.

Year	R	information content Values						Weights Values					
		PR	DR	RR	HS	CA	RE	PR	DR	RR	HS	CA	RE
** 2019**	** WA**	0.308	0.417	0.287	0.500	0.367	0.325	0.140	0.189	0.130	0.227	0.167	0.147
**2021**		0.345	0.465	0.393	0.473	0.435	0.391	0.138	0.186	0.157	0.189	0.174	0.156
**2019**	**LIC & LMC**	0.172	0.305	0.208	0.244	0.221	0.104	0.137	0.243	0.166	0.195	0.176	0.083
**2021**		0.472	0.448	0.324	0.230	0.206	0.129	0.261	0.248	0.179	0.127	0.114	0.071
**2019**	**UMC**	0.176	0.279	0.190	0.444	0.233	0.271	0.110	0.175	0.119	0.279	0.146	0.170
**2021**		0.176	0.296	0.165	0.366	0.185	0.316	0.117	0.197	0.110	0.243	0.123	0.210
**2019**	**HIC**	0.231	0.565	0.289	0.432	0.351	0.168	0.113	0.277	0.142	0.212	0.172	0.083
**2021**		0.311	0.599	0.350	0.400	0.483	0.179	0.134	0.258	0.151	0.172	0.208	0.077

Note: R: Region; WA: western Asia astern; LIC: low-income countries; LMC: Lower-middle-income countries; UMI: Upper-middle-income countries; HIC: high-income countries PR: prevention; DR: detection and reporting; RR: rapid response; HS: health system; CA: commitments and adherence; RE: risk environment.

### 4.2 Health security performance ranking and clustering results

Table 2 summarizes the results of a COCOSO analysis and K-means clustering on the health security performance of WA countries for 2019 and 2021. Countries are divided into LIC, LMC, UMC and HIC groups. Key metrics include the health security score ( *C*_*i*_), regional rank ( *R*_*i*_), cluster membership ( *S*_*i*_), and changes in scores and ranks over the two years. Higher *C*_*i*_ and *R*_*i*_ indicate better performance, while cluster membership ranges from Cluster 1 (best) to Cluster 5 (worst).

**Table 2.** Health security performance ranking and clustering results.

	Country		2019			2021			Shifts		
		type	Ki	Ri	Si	Ki	Ri	Si	Ki	Ri	Si
***1***	*Bahrain*	*HIC*	*8.816*	*11*	*3*	*5.407*	*14*	*3*	*-3.409*	*-3*	*0*
***2***	*Cyprus*	*HIC*	*9.729*	*8*	*3*	*6.694*	*8*	*2*	*-3.035*	*0*	*1*
***3***	*Israel*	*HIC*	*12.940*	*2*	*2*	*7.537*	*5*	*2*	*-5.402*	*-3*	*0*
***4***	*Kuwait*	*HIC*	*9.232*	*9*	*3*	*6.009*	*11*	*3*	*-3.222*	*-2*	*0*
***5***	*Oman*	*HIC*	*9.075*	*10*	*3*	*6.216*	*9*	*3*	*-2.859*	*1*	*0*
***6***	*Qatar*	*HIC*	*10.917*	*6*	*2*	*7.980*	*4*	*1*	*-2.937*	*2*	*1*
***7***	*Saudi Arabia*	*HIC*	*10.987*	*5*	*2*	*7.257*	*6*	*2*	*-3.731*	*-1*	*0*
***8***	*United Arab Emirates*	*HIC*	*8.481*	*12*	*4*	*6.216*	*10*	*3*	*-2.265*	*2*	*1*
***9***	*Syria*	*LIC*	*0.972*	*17*	*5*	*1.906*	*16*	*5*	*0.935*	*1*	*0*
***10***	*Yemen*	*LIC*	*1.310*	*16*	*5*	*0.911*	*17*	*5*	*-0.399*	*-1*	*0*
***11***	*Jordan*	*LMC*	*9.841*	*7*	*3*	*7.118*	*7*	*2*	*-2.723*	*0*	*1*
***12***	*Lebanon*	*LMC*	*7.731*	*13*	*4*	*5.626*	*12*	*3*	*-2.106*	*1*	*1*
***13***	*Armenia*	*UMC*	*16.341*	*1*	*1*	*9.635*	*1*	*1*	*-6.706*	*0*	*0*
***14***	* Azerbaijan*	*UMC*	*6.892*	*14*	*4*	*5.519*	*13*	*3*	*-1.373*	*1*	*1*
***15***	*Georgia*	*UMC*	*11.298*	*4*	*2*	*8.311*	*2*	*1*	*-2.987*	*2*	*1*
***16***	*Iraq*	*UMC*	*3.269*	*15*	*5*	*3.740*	*15*	*4*	*0.470*	*0*	*1*
***17***	*Turkey*	*UMC*	*12.589*	*3*	*2*	*8.065*	*3*	*1*	*-4.525*	*0*	*1*

### 4.3 The relationships between independent financial resource allocation indicators and D-CRITIC-COCOSO Health Security Outcomes

Our study used the Spearman rank correlation method to analyse the relationship between financial resource allocation indicators, specifically financing for health security initiatives (F1) and public healthcare spending per capita (F2), with overall health security performance scores (F3) across 17 countries. We found strong, positive correlations between both financial indicators and health security performance, with correlation coefficients of 0.98 for F1 and F3 and 0.97 for F2 and F3. These findings suggest a substantial association between financial investment in health security and the capacity to prevent, detect, and respond effectively to health threats.

### 4.4 Discussion

***4.4.1 Health security priorities in western Asia (2019-2021)***

Our analysis reveals a consistent emphasis on strengthening foundational health infrastructure, highlighted by the sustained high weight of the Health System (HS) indicator, which received weights of 0.227 in 2019 and 0.189 in 2021. This underscores the robust commitment to developing a resilient healthcare system capable of addressing diverse health challenges. These findings align with the ASPR’s National Health Security Strategy (2019–2022), which emphasizes the necessity of a strong health system to manage emerging health threats and to enhance resilience against future crises ( ASPR, 2019). The significant weight assigned to the Detection and Reporting (DR) indicator further illustrates the importance of timely and accurate information in health security. With weights of 0.189 in 2019 and 0.186 in 2021, this underscores the critical role of robust disease surveillance and reporting systems in mitigating public health emergencies, resonating with the U.S. government’s Global Health Security Strategy ( David, 2024). While foundational health infrastructure and detection/reporting mechanisms remain central to Western Asia’s health security strategy, a noteworthy shift towards prioritizing rapid response (RR) and risk environment (RE) management has emerged in 2021. This change reflects the dynamic nature of health security challenges, as illustrated by the COVID-19 pandemic, which necessitates adaptive policymaking ( Roasio et al., 2022).

The third-ranking indicator, commitments and adherence (CA), also gained prominence, with weights of 0.167 and 0.174 in 2019 and 2021, respectively. This trend indicates that policymakers are increasingly recognizing the importance of maintaining health protocols and international commitments as foundational for effective health security. Conversely, indicators such as RE, Prevention (PR), and RR initially exhibited lower weights, suggesting that they were deprioritized in favor of foundational elements. However, the increased weights for RR (0.157) and RE (0.156) in 2021 indicate a strategic pivot towards enhancing preparedness for immediate health crises and acknowledging the impacts of environmental health risks.

This adaptive approach in health security policymaking is further supported by studies highlighting the effectiveness of Rapid Response Teams (RRTs) in reducing mortality rates and improving patient outcomes in critical situations ( Alves Silva et al., 2021). For instance, a systematic review demonstrated that RRT implementation significantly decreased the incidence of cardiac arrest and overall mortality in hospitals, emphasizing the essential role of timely intervention during emergencies. Additionally, the increased emphasis on RE reflects a growing awareness of how environmental factors can exacerbate health crises, as outlined by the Asia-Pacific Health Security Action Framework ( World Health Organization, 2024), which advocates for multispectral strategies to strengthen resilience against public health emergencies. Our findings suggest that resource allocation should continue to prioritize foundational health systems and robust detection/reporting capabilities while also addressing the evolving need for effective rapid response mechanisms and comprehensive environmental risk management. Moreover, this strategic shift toward a more adaptive health security strategy addresses gaps identified by the Global Health Security Index (GHSI), which highlights deficiencies in public health emergency response plans in several countries ( Bellm & Nuzzo, 2021). By prioritizing foundational infrastructure, detection/reporting, rapid response, and risk environment management, Western Asia is making strides to enhance its overall health security. The dynamic nature of health threats, especially as highlighted by the COVID-19 pandemic, necessitates an adaptive approach to health security policymaking ( Roasio et al., 2022). The increased weights for RR and RE indicate a dual focus on responding to immediate crises and preparing for the future challenges posed by infectious diseases and environmental hazards. This aligns with Nasser and Alghawli’s (2024) assertion that effective decision-making requires continuous evaluation and comparison. By focusing on these key indicators, policymakers can significantly enhance health security outcomes, ultimately fostering a more resilient public health landscape in Western Asia.

***4.4.2 Health security priorities across income groups***

Table 1 and Figures 1 and 2 showcase the changes in D-CRITIC weightings of health security indicators for different income groups (HIC, UMC, LIC/LMC) from 2019 to 2021. High-income countries (HICs) consistently prioritized detection and reporting (DR), health systems (HS), and commitment and adherence (CA) in both years. In 2019, DR led to 0.277, followed by HS at 0.212 and CA at 0.172. By 2021, DR remains the top priority at 0.258, CA increases to 0.208, and HS decreases slightly to 0.172. This minor shift indicates slight reprioritization, with CA gaining prominence. Prevention scores remained consistently low, suggesting that this was not a primary concern for HICs.

Upper-middle-income countries (UMCs) focus on health systems (HS), detection and reporting (DR), and risk environments (RE) as their main priorities. In 2019, HS had the highest weighting at 0.279, followed by DR at 0.175 and RE at 0.170. A notable shift occurred in 2021, with RE gaining importance and increasing to 0.210, surpassing DR at 0.197. This change likely reflects an increased awareness of environmental and external risks. HS remained a priority despite a slight decrease in weighting, whereas commitments and adherence continued to rank relatively low, similar to HICs.

Low- and lower-middle-income countries (LICs/LMCs) have experienced a significant shift from 2019 to 2021. In 2019, DR (0.243), HS (0.195), and CA (0.176) were the focus. However, by 2021, prevention (PR), DR, and rapid response (RR) have emerged as top-weighted indicators. PR showed a substantial increase to 0.261, with a DR of 0.248 and an RR of 0.179. This change indicates a greater emphasis on proactive health security measures, with prevention becoming the primary focus. The lower weighting for HS may suggest resource limitations or the prioritization of other immediate needs over structural healthcare improvements.

Across all income groups, DR consistently received high weights, highlighting a universal priority for early health threat identification. HS showed a general decline in weighting over time, particularly in LICs and LMCs, possibly due to resource constraints that limit structural investments. The significant increase in the prevention of LICs and LMCs by 2021 may reflect the growing focus on preventive healthcare measures. These findings indicate evolving health security priorities, with LICs and LMCs increasingly emphasizing prevention, whereas HICs and UMCs focus on detection capabilities. This shift may be influenced by region-specific challenges and capacity differences, especially in response to the post-2019 health security events.

***4.4.3 Ranking and clustering results***

This study’s regional analysis across Western Asia (WA) from 2019 to 2021 reveals important trends in health security, particularly as countries faced the COVID-19 pandemic’s far-reaching impacts. Results show a mixed picture: while a few countries improved or maintained their positions within their clusters, others experienced notable declines, underscoring persistent health security gaps across the region. These findings highlight the effects of shared vulnerabilities, resource constraints, and the role of international partnerships in bolstering public health systems. The implications of these results span clusters and point to strategic areas for policy intervention, targeted resource allocation, and regional collaboration.

• Cluster 1: High Performers with Relative Declines

**Figure 3.**
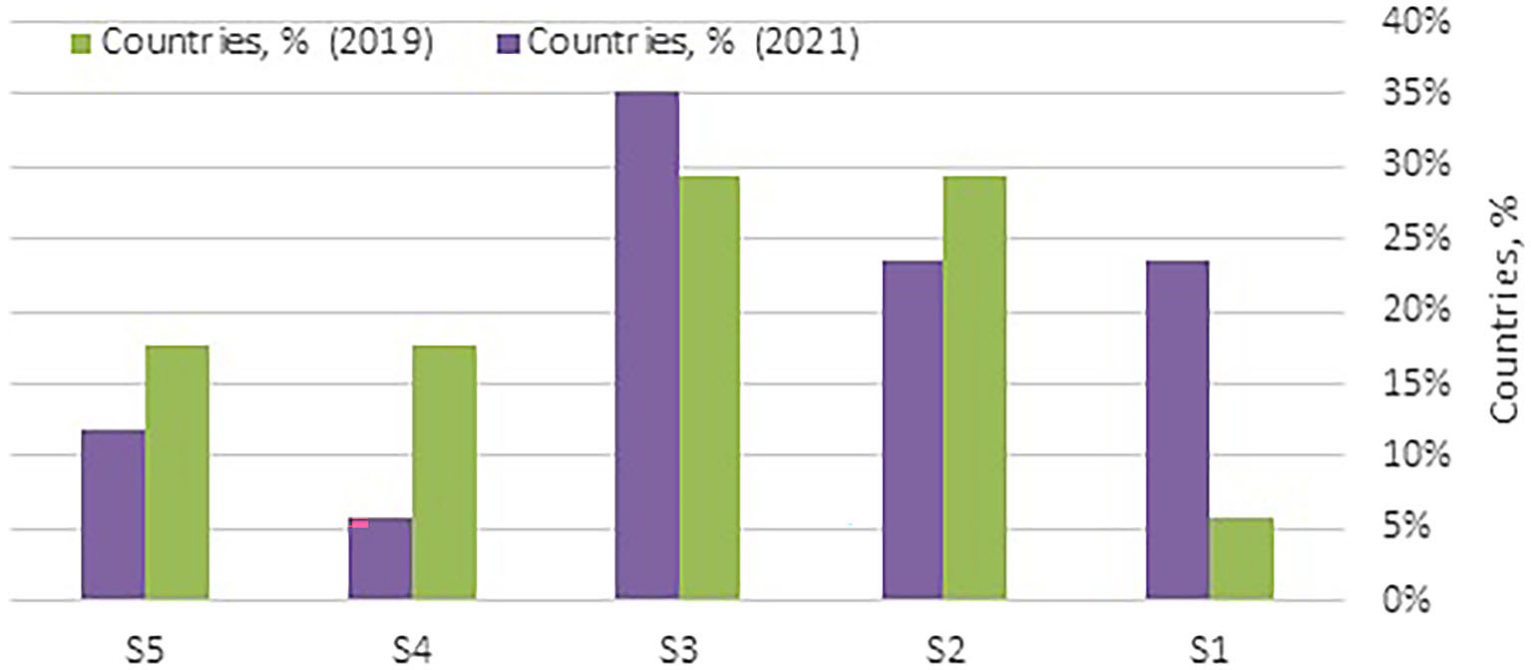
Distribution of WA countries by their clusters. (Source: Authors). This bar chart illustrates the distribution of Western Asia (WA) countries across clusters (S1 to S5) in 2019 and 2021. The clusters are categorized based on health security performance, where Cluster 1 (S1) represents excellent performance and Cluster 5 (S5) indicates poor performance. This visualization highlights changes in the health security status of WA countries over the specified years, offering insights into regional progress and disparities.

As shown in Figure 3, countries in Cluster 1, representing the highest health security performers in WA,, display trends that reveal both resilience and vulnerability in the face of regional pressures. Armenia, for instance, experienced a substantial decline in its Composite Index (Ci) score, dropping from 16.341 in 2019 to 9.635 by 2021. This significant reduction, attributed to internal challenges and exacerbated by pandemic-related constraints, mirrors findings by Zhang et al. (2021), who noted similar declines in regional health resilience under shared resource pressures ( Zhang et al., 2021). Armenia’s retention of its top rank within WA, despite this decline, suggests that other countries in the region similarly struggled with pandemic challenges and that the gap between higher and lower performers may be widening.

In contrast, Qatar’s health security investments yielded an improved regional ranking, moving from 6th to 4th between 2019 and 2021. Qatar’s progress highlights the role of high-income countries (HIC) in leveraging resources for health system reforms, as shown by Ravi et al. (2020), who documented how targeted investments enhance resilience and crisis response ( Ravi et al., 2020). Georgia’s ascension to the second rank within Cluster 1 demonstrates the critical impact of sustained international partnerships, specifically its collaboration with the CDC, which has supported Georgia’s health infrastructure over decades ( Biggs, 2020; Lal et al., 2022). This finding underscores the need for continuous external support to strengthen regional health security, especially for countries that lack the resources to maintain advanced health infrastructure independently.

• Cluster 2: Incremental Improvements in Health Capacity

Analysis of Cluster 2 countries shows modest but notable improvements, particularly in detection (DR) and health system capacity (HS) metrics. For instance, Jordan and Cyprus moved from the third to the second cluster, although their regional rankings remained stable at seventh and eighth, respectively. Jordan’s 19% improvement in DR and 17% increase in HS scores underscore the role of digital health investments, including telemedicine, in bolstering health capacities during the COVID-19 pandemic, as Alsabi et al. (2023) demonstrated ( Alsabi et al., 2023). Similarly, Cyprus’s gains in DR and HS indicate that even modest investments in health technology and infrastructure can have significant impacts on national health security.

Despite these improvements, other high-income countries (HICs) in Cluster 2, such as Israel and Saudi Arabia, experienced slight declines in their regional rankings, falling from 2nd to 5th and 5th to 6th, respectively. This suggests that without sustained investment and regional cooperation, even high-income nations can see stagnation or regression in health security performance. Regional collaboration on health infrastructure, data sharing, and workforce development could provide a more stable foundation for health security in the region, as emphasized in studies on health resilience in high-income nations facing systemic constraints ( Binagwaho et al., 2022).

• Cluster 3: Variable Trends and Rising Health System Needs

The countries in Cluster 3 reveal both improvements and challenges. An increase in the number of WA countries from five (29%) in 2019 to six (35%) in 2021, as shown in Figure 3, reflects a slight increase in health security needs in this cluster, where most nations are either high-income (67%), lower-middle-income (LMC) (17%), or upper-middle-income (UMC) (17%). Oman’s improvement from the 10th to the 9th regional rank demonstrates incremental progress, likely supported by targeted investments in health capacity and detection infrastructure. Conversely, Bahrain and Kuwait experienced minor declines in their rankings, highlighting the need for continued investment in preventive and rapid response capabilities to sustain health security gains.

Lebanon and Azerbaijan’s advancement in regional and cluster rankings suggests that even under economic constraints, countries can improve health security by prioritizing critical areas, such as prevention (PR) and detection (DR). Research indicates that optimizing resource allocation towards these core functions, especially in times of crisis, can significantly improve a country’s health system resilience ( Kruk et al., 2017). The trends in Cluster 3 suggest that WA countries could benefit from a model that emphasizes foundational health security investments even in the absence of extensive resources to ensure sustainability and incremental progress.

• Clusters 4 and 5: Persistent Vulnerabilities in Low-Resource Nations

The most concerning findings relate to Clusters 4 and 5, where low-income and conflict-affected countries, such as Iraq, Yemen, and Syria, have faced regressions or stagnation in health security capabilities. Despite modest increases in certain health scores, Iraq’s decline from the fourth to the fifth cluster indicates insufficient systemic resilience to maintain previous gains. Iraq’s lower detection (DR) and prevention (PR) scores indicate gaps in disease surveillance and public health infrastructure; areas that are crucial for crisis response are often neglected in low-resource settings ( Hamalaw et al., 2021).

Syria and Yemen’s consistent positioning in the fifth cluster reveals systemic public health weaknesses exacerbated by ongoing political and economic instability. Yemen’s PR score of just 0.8 in 2021 underscores the severe limitations in preventive health capabilities, leaving the country highly vulnerable to disease outbreaks. Similarly, Syria’s low DR and HS scores illustrate critical deficiencies in early detection and health-system capacity, which is consistent with studies noting that crisis-affected regions require a comprehensive approach that combines infrastructure development, workforce training, community engagement, and policy reform to achieve long-term health security ( Alhaffar & Janos, 2021).

***4.4.3 Spearman rank correlation results***

The near-perfect correlations between financial resource allocation indicators, specifically financing for health security initiatives (F1) and public healthcare spending per capita (F2), with overall health security performance scores (Ci) across WA countries, underscore that financial investment plays a critical role in bolstering health security. Higher levels of both targeted health security financing (F1) (0.98) and general public healthcare spending (F2) (0.97) were consistently associated with higher health security performance (F3). This relationship is particularly relevant for countries with substantial financial inputs, such as Armenia and Georgia, which show high health security scores, reflecting the effectiveness of financial investments in achieving improved preparedness and response capabilities. Conversely, countries with minimal or no spending in these categories, such as Syria and Yemen, exhibit some of the lowest health security scores, highlighting a likely resource gap in public health infrastructure that limits their resilience against health threats.

### 4.5 Implications and recommendations

The observed trends indicate shifting priorities in health security strategies tailored to resource levels and specific risks faced by different income groups. High-income and upper-middle-income countries continue to prioritize detection and reporting, emphasizing the early identification of health threats to support rapid responses. By 2021, low- and lower-middle-income countries are increasingly focusing on prevention as a top priority, reflecting a proactive approach in contexts where reactive capacities may be limited. The lower weighting for health systems across all groups suggests either resource limitations or strategic reallocation toward other critical health security areas, possibly in response to post-2019 health events. These evolving priorities underscore diverse approaches to health security, reflecting each income group’s unique challenges, resources, and public health goals.

Furthermore, this study highlights considerable disparities in health resilience among countries shaped by income levels, resource availability, and political stability. High-income countries (HICs), such as Qatar and Georgia, improved their health security rankings by leveraging financial investments and strategic international partnerships, while conflict-affected, low-resource nations, namely Iraq, Yemen, and Syria, experienced stagnation or decline. These findings underscore the urgent need for a regional framework to address health security inequities, bridge the gap between high- and low-income nations, and foster sustainable public health resilience across the WA. The observed disparities align with the broader global patterns seen in low-income and politically unstable regions, where limitations in funding and infrastructure often constrain health security progress. Iraq’s mixed performance—with some improvements in detection and reporting (DR) but an overall cluster decline—illustrates the challenge of sustaining health system gains in volatile settings without consistent, comprehensive investment and policy commitment. Similarly, Yemen’s persistently low scores in prevention (PR) mirror challenges observed in sub-Saharan Africa, where under-resourced health systems struggle to prioritize preventive health measures, leading to high public health burdens and limited security capabilities ( Nasser & Alghawli, 2024).

The results indicated that the challenges faced by Iraq, Yemen, and Syria in health security are intricately linked to substantial resource allocation issues. For Iraq, the decline from the fourth to the fifth cluster, despite some advances in specific areas, such as detection, suggests that targeted investments without a holistic, integrated approach may be insufficient. Improvements in specific functions such as detection capabilities offer limited, temporary benefits if not supported by robust health infrastructure, workforce training, and preventive care, which are necessary for long-term resilience ( Emami et al., 2024). The persistently low PR and DR scores for Yemen and Syria emphasize the urgent need for foundational health infrastructure investments, particularly in crisis-prone regions where preventive measures are essential to mitigate disease spread. Research in crisis settings highlights that strengthening early detection and preventive health measures reduces the spread of communicable diseases and alleviates public health burden over time ( Meckawy et al., 2022). Allocating resources to these core health security areas, such as early intervention, surveillance, and effective disease management, can enhance health resilience and reduce vulnerability to future crises.

The findings of the correlation analysis have important policy implications. First, they suggested that investments in health security financing (F1) and general healthcare spending (F2) are likely critical for achieving higher health security performance. Governments and international organizations aiming to enhance global health security might consider these financial indicators as key benchmarks for strengthening the health system. By strategically increasing F1 and F2, countries may improve their ability to meet international health security standards and enhance their response capabilities in line with the Joint External Evaluation (JEE) and Performance Verification System (PVS) recommendations. To mitigate the identified challenges, policy interventions that prioritize preventive care, detection, and overall health system resilience are crucial for countries facing stagnation or decline in health security. For Iraq, policy adjustments focused on integrating resources across crisis responses, infrastructure, and workforce development could promote a balanced health system and ensure sustainable improvement. For Yemen and Syria, persistently low PR and DR scores underscore the need for both regional and international support to establish a basic public health infrastructure. Regional cooperation can also standardize protocols, enhance healthcare workforce training, and align public health policies with international standards, thus enabling lower-resource countries to manage public health threats more effectively and reduce their vulnerability to crises.

In summary, the findings of this study highlight the essential need for a unified regional approach to health security in WA, encouraging equitable resource allocation, coordinated health strategies, and comprehensive policy interventions that collectively enhance public health resilience across various economic and political landscapes.

## 5. Conclusion

This study provides a comprehensive assessment of health security performance across Western Asia (WA) from 2019 to 2021, revealing both progress and persistent vulnerabilities within the region. A significant finding from this study is the critical role of foundational health infrastructure, particularly in areas such as health system capacity (HS), detection and reporting (DR), rapid response (RR), and environmental risk management (RE). The consistently high weighting of the HS and DR indicators underscores their importance in achieving resilient health systems capable of mitigating public health threats. However, the increased emphasis on RR and RE in 2021 reflected an adaptive shift in health security priorities following the COVID-19 pandemic. This shift suggests that while stable health systems and robust detection mechanisms are essential, there is also a growing need for effective rapid response measures and environmental risk management to address immediate and evolving crises.

The findings highlight significant disparities in health security shaped by variations in income levels, political stability, and resource availability. High-income countries (HICs), such as Qatar and Georgia, demonstrated the benefits of strategic investments and international partnerships, which enabled them to improve their health security rankings despite regional challenges. Conversely, conflict-affected, low-resource nations, such as Iraq, Yemen, and Syria, faced stagnation or even regression, illustrating the difficulties of sustaining health security gains without consistent resources and support.

The cluster analysis illustrates that while some countries in WA managed incremental improvements in health security, others experienced setbacks, widening the gap between high- and low-performing nations. The decline of Iraq, Yemen, and Syria to lower clusters reveals the limitations of targeted short-term investments when not supported by a holistic and integrated approach. In these countries, challenges in resource allocation have hindered the capacity to develop comprehensive health systems. The low scores in prevention (PR) and detection/reporting (DR) highlight the urgent need for a foundational infrastructure that facilitates early detection and effective disease management. This trend aligns with global findings in similar low-resource settings, where limited preventive measures and weak surveillance systems increase the vulnerability to health crises.

Our analysis also demonstrated a significant positive association between financial investments in health security and public healthcare spending and overall health security performance. The strong correlations suggest that increased funding in these areas could substantially enhance a country’s health security capabilities, thereby improving its resilience to public health threats. As global health risks continue to evolve, these findings advocate targeted financial investments as part of comprehensive health-security strategies.

This study’s implications point to the necessity of a unified regional health security framework in WA that fosters equitable resource sharing, coordinated health strategies, and robust international partnerships. Through the consolidation of resources and expertise, Western African countries can address disparities in health resilience more effectively, particularly in nations facing significant economic or political challenges. Regional cooperation has the potential to standardize health protocols, enhance healthcare workforce training, and align policies with international standards, thereby strengthening health security across the region. Furthermore, the findings of this study underscore the critical need for coordinated efforts to bridge gaps in health security and to position the region to foster a robust and sustainable public health landscape.

### Glossary

**CoCoSo** (The Combined Compromise Solution): A MCDM ranking model.

**D-CRITIC (Distance Correlation-based Criteria Importance through Inter-criteria Correlation):** A method for determining the relative importance of criteria.

**Global Health Security Index (GHSI)**: A comprehensive assessment tool that measures health security capabilities across countries, focusing on various indicators that contribute to national and global health security.

**Multi-Criteria Decision Making (MCDM)**: A set of methods and techniques used to evaluate and prioritize multiple conflicting criteria in decision-making processes.

**WA** (Western Asia) A geographical region in Asia.

### Ethics and consent

No Ethical approval or consent needed.

## Author contributions

Adel A. Nasser: Conceptualization, methodology, formal analysis, software, writing—original draft preparation; Abed Saif Ahmed Alghawli: Conceptualization, methodology, formal analysis, funding acquisition, and writing—original draft preparation; S. Saleh: Data curation, validation, investigation, resources, writing—review and editing, supervision; Amani A. K. Essayed: Visualization, software, and writing—review and editing.

## Declaration of generative AI and AI-assisted technologies in the writing process

During the preparation of this work the author(s) used [paperpal, quillbot, aistudio, and ChatGPT] for language refinement and structure. After using this tools, the author(s) reviewed and edited the content as needed and take(s) full responsibility for the content of the publication.

## Data Availability

The data supporting the findings of this study are publicly available and can be accessed through the following repository ( Global Health Security Index, Global Health Security Index Data Model and Report. (2021), at https://ghsindex.org/report-model/), ( Global Health Security Index, 2021). 1. Figshare: Supplementary File 1- Income-Based Analysis of Health Security in Western Asia through an Integrated GHSI, MCDM, and Clustering Model.pdf. Comprehensive data can be found in Tables 1-3 of the supplementary file **Doi**: https://doi.org/10.6084/m9.figshare.27992735.v3 ( Nasser, et al. 2024b) This project contains the following extended data:
• Supplement File 1.pdf Data are available under the terms of the CC BY 4.0
2. Zenodo: supplementary-material-and-softwares: 1.0.0 Doi: https://doi.org/10.5281/zenodo.14541236 ( Nasser, et al. 2024b). The data supporting the findings of this study are publicly available and can be accessed through the following repository ( https://github.com/ProfAdelAbdulsalam/supplementary-material-and-softwares/tree/1.0.0 )
• This project contains the following extended data: ProfAdelAbdulsalam-supplementary-material-and-softwares-37cf50a
○ LICENSE ○ README.md ○ Supplement File 1- Distance Correlation-based CRITIC software1.xlsx ○ Supplement File 1-Supplement File - Income-Based Analysis of Health Security in Western Asia through an Integrated GHSI, MCDM, and Clustering Model.pdf ○ Supplementary Software 2-An Integrated GHSI, MCDM, and Clustering Model for Health Security Analysis.xlsm All data, processing and analysis results, figures, and tables related to this study are presented in Supplementary software 2 ( Nasser et al., 2024b). This file integrates the processes of weighting, ranking, clustering, and Spearman rank correlation analyses into a single Excel-based tool, offering a comprehensive framework for evaluating the health security performance of Western Asian nations across all income groups (LIC, LMC, UMC, HIC) for the years 2019 and 2021. Source software available from: (( https://github.com/ProfAdelAbdulsalam/supplementary-material-and-softwares/tree/1.0.0)). Archived software available from ( https://doi.org/10.5281/zenodo.14541236 ) ( Nasser, et al., 2024b). License: OSI approved open license software is under GNU General Public License v3.0). This supplementary resource provides detailed support for replicating the study’s methods and results.
