# Supplementary material for: Income-Based analysis of health security in Western Asia through an integrated GHSI, MCDM, and Clustering Model
Source: F1000Res. 2025 Mar 28;14:43. Originally published 2025 Jan 8. [Version 2] doi: 10.12688/f1000research.159002.2 (PMC11923535; doi:10.12688/f1000research.159002.2)
Supplement: Peer review history

# Reviewer report: Reviewer response for version 1

**DOI:** 10.5256/f1000research.174669.r367984

**Published:** 2025-03-17

**Authors:** Prof Ahmed Sultan Al-Hegami

**Affiliations:** 1 Sana'a University, Sana'a, Yemen

The paper provides a thorough evaluation of health security performance in Western Asia (WA), highlighting both advancements and ongoing challenges in the region. A notable strength of the study is its use of cluster analysis, which allows for a clear depiction of how various countries within WA have progressed differently. The findings indicate that while some nations have made incremental improvements, others—specifically Iraq, Yemen, and Syria—have regressed, exacerbating disparities in health security performance. This observation serves as a crucial reminder of the limitations inherent in short-term investments that lack a comprehensive and integrated strategy.

 The paper’s insights are particularly relevant for policymakers and stakeholders in the region, as it emphasizes the need for sustained and holistic approaches to health security. Overall, the study contributes valuable knowledge to the field, urging a shift toward more robust and interconnected health systems to better prepare for future challenges.

 Here are some recommendations and observations for enhancing the manuscript:

 Comment 1: I recommend summarizing the main contributions of the study more clearly. For instance, the introduction of an integrated MCDM and K-means framework is significant, as it combines multicriteria decision-making with dynamic clustering to address the limitations of global indices like the GHSI. This enables a nuanced analysis of health security in Western Asia (WA). Additionally, the study highlights the divergent priorities among WA regions, emphasizing the need for context-specific policy approaches. It effectively maps health security priorities and classifies countries, revealing intra-regional disparities and gaps in post-pandemic resilience. Lastly, the identification of gaps, along with data-driven recommendations, will be crucial for the ongoing development of health security in WA.

 Comment 2. I recommend that the authors expand their discussion to better illustrate the implications of their findings using the methodology for enhancing health security in WA countries and shaping global health governance. For example, elaborate on how countries can prioritize investments and resource allocation. Provide more detail on tailored interventions for each cluster, emphasizing practical implementation and the rationale behind these strategies.

 Comment 3: Suggest areas for future research that could build on the findings.

Is the work clearly and accurately presented and does it cite the current literature?

Yes

If applicable, is the statistical analysis and its interpretation appropriate?

I cannot comment. A qualified statistician is required.

Are all the source data underlying the results available to ensure full reproducibility?

Yes

Is the study design appropriate and is the work technically sound?

Yes

Are the conclusions drawn adequately supported by the results?

Yes

Are sufficient details of methods and analysis provided to allow replication by others?

Yes

Reviewer Expertise:

My research interest includes Artificial Intelligence, Data Mining, Machine Learning, Big Data, Data Science, Intelligent Business ,Temporal Databases, Decision Support Systems, Real Time Systems and Knowledge Discovery in Databases.

I confirm that I have read this submission and believe that I have an appropriate level of expertise to confirm that it is of an acceptable scientific standard.

# Response

**Published:** 2025-03-17

**Authors:** Abed Saif Ahmed Alghauli

**Affiliations:** Computer Science, Prince Sattam bin Abdulaziz University, Al Kharj, Riyadh Province, Saudi Arabia

**Responses to Peer Review Report of Prof. Ahmed Sultan Al-Hegami, Sana'a University, Sana'a, Yemen**

**Dear Prof. Ahmed Sultan Al-Hegami,**

 Thank you for taking the time to review our manuscript and for your valuable feedback. Your insights have been instrumental in improving the quality of our work.

**Comment 1:** I recommend summarizing the main contributions of the study more clearly. For instance, the introduction of an integrated MCDM and K-means framework is significant, as it combines multicriteria decision-making with dynamic clustering to address the limitations of global indices like the GHSI. This enables a nuanced analysis of health security in Western Asia (WA). Additionally, the study highlights the divergent priorities among WA regions, emphasizing the need for context-specific policy approaches. It effectively maps health security priorities and classifies countries, revealing intra-regional disparities and gaps in post-pandemic resilience. Lastly, the identification of gaps, along with data-driven recommendations, will be crucial for the ongoing development of health security in WA.

**Response:** Thank you for your valuable feedback. In response, we have added a new paragraph at the end of the Introduction that succinctly summarizes the significant contributions of our study. This includes the introduction of the integrated MCDM and K-means framework, the analysis of intra-regional disparities, and the practical recommendations provided for enhancing health security in WA.

**Comment 2:** I recommend that the authors expand their discussion to better illustrate the implications of their findings using the methodology for enhancing health security in WA countries and shaping global health governance. For example, elaborate on how countries can prioritize investments and resource allocation. Provide more detail on tailored interventions for each cluster, emphasizing practical implementation and the rationale behind these strategies.

**Response:** Thank you for your constructive feedback. In response, we have revised the first section, "4.5 Implications and Recommendations." We have added a new paragraph at the end of this section to emphasize the practical implications of our study from two perspectives: improving health security in WA and advancing the Sustainable Development Goals (SDGs). This includes specific strategies for prioritizing resource allocation and tailored interventions for each cluster, with an emphasis on practical implementation and the rationale supporting these strategies.

**Comment 3:** Suggest areas for future research that could build on the findings.

**Response:** Thank you for your insightful comment regarding the inclusion of a limitations and future work section. We recognize the importance of addressing the constraints of our study and outlining potential avenues for further research. In response to your feedback, we have added a dedicated section that discusses the limitations and proposes future research directions. This section outlines how future studies can build upon our findings, including exploring alternative methodologies, expanding the dataset, and examining additional regional contexts to enhance the robustness of health security analyses.

 We hope that these responses adequately address your concerns and further strengthen our manuscript.

 Thank you again for your valuable insights!

# Reviewer report: Reviewer response for version 1

**DOI:** 10.5256/f1000research.174669.r358024

**Published:** 2025-01-17

**Authors:** Laith R. Flaih

**Affiliations:** 1 Cihan University-Erbil, Kurdistan Region, Iraq

Referee's Report

 To: F1000Research Journal

 Title of Paper: Income-Based    analysis of health security in Western Asia through an integrated GHSI, MCDM, and cloistering model.

 Authors: Naser A. A., Alghawli ASA, Saleh S. and Elsayed AAK.

 Overall

 In general, the manuscript contains many good results and is considered a good scientific addition to the field of study in which a novel approach was used.

 Reviewer Comments

 The following are suggestions and comments for improving the manuscript:

 1. Add novelty of this work at the end of introduction section.

 2. The justification for using k-means clustering could be elaborated.

 3. Add the study limitations section

 4. While the manuscript is generally well written, minor issues should be revised, for example, in a conclusion section “Western African countries can address disparities in health resilience more effectively……."

 Recommendation 

 I recommend accepting the paper with minor revisions to address the comments outlined above. The proposed enhancements will improve the manuscript’s clarity, accessibility, and impact, ensuring it meets the high standards of your journal.

 Best regards,

 Dr. Laith R.

Is the work clearly and accurately presented and does it cite the current literature?

Yes

If applicable, is the statistical analysis and its interpretation appropriate?

Yes

Are all the source data underlying the results available to ensure full reproducibility?

Yes

Is the study design appropriate and is the work technically sound?

Yes

Are the conclusions drawn adequately supported by the results?

Yes

Are sufficient details of methods and analysis provided to allow replication by others?

Yes

Reviewer Expertise:

Security, Big data analysis

I confirm that I have read this submission and believe that I have an appropriate level of expertise to confirm that it is of an acceptable scientific standard.

# Response

**Published:** 2025-03-17

**Authors:** Abed Saif Ahmed Alghauli

**Affiliations:** Computer Science, Prince Sattam bin Abdulaziz University, Al Kharj, Riyadh Province, Saudi Arabia

**Responses to Peer Review Report of Laith R. Flaih, Cihan University-Erbil, Kurdistan Region, Iraq**

**Dear Prof. Laith R. Flaih,**

 Thank you for your thoughtful review and for the insightful comments provided on our manuscript. Your feedback has been invaluable in guiding our revisions.

**Comment 1:** Add novelty of this work at the end of the introduction section.

**Response:** Thank you for your valuable feedback. In response, we have added a new paragraph at the end of the Introduction that highlights the significant contributions and novelty of our study.

**Comment 2:** The justification for using K-means clustering could be elaborated.

**Response:** Thank you for your valuable feedback regarding Section 2.4. We appreciate your suggestion to elaborate on the justification for using K-means clustering. In response, we have updated this section to provide a more in-depth discussion of the rationale behind selecting K-means as our clustering method

**Comment 3:** Add the study limitations section.

**Response:** Thank you for your insightful comment regarding the inclusion of a limitations and future work section. We recognize the importance of addressing the constraints of our study and outlining potential avenues for further research. In response to your feedback, we have added a dedicated section that discusses the limitations and proposes future research directions.

**Comment 4:** While the manuscript is generally well written, minor issues should be revised, for example, in the conclusion section “Western African countries can address disparities in health resilience more effectively…”

**Response:** Thank you for your thoughtful review and for pointing out the minor issues in the manuscript. We appreciate your attention to detail and have made the necessary corrections to the conclusion section. The reference to "Western African countries" has been updated to "Western Asian" to ensure accuracy and clarity in our findings

 We hope that these responses adequately address your concerns and further improve our manuscript. Thank you again for your valuable insights!
